# Supplementary material for: Transcriptomics in serum and culture medium reveal shared and differential gene regulation in pathogenic and commensal Streptococcus suis
Source: Microb Genom. 2023 Apr 27;9(4):mgen000992. doi: 10.1099/mgen.0.000992 (PMC10210958; doi:10.1099/mgen.0.000992)
Supplement: Supplementary material 4 [file mgen-9-992-s001.pdf]

Supplementary figures

Transcriptomics in serum and culture medium reveal shared and differential gene regulation in pathogenic and commensal *Streptococcus suis*

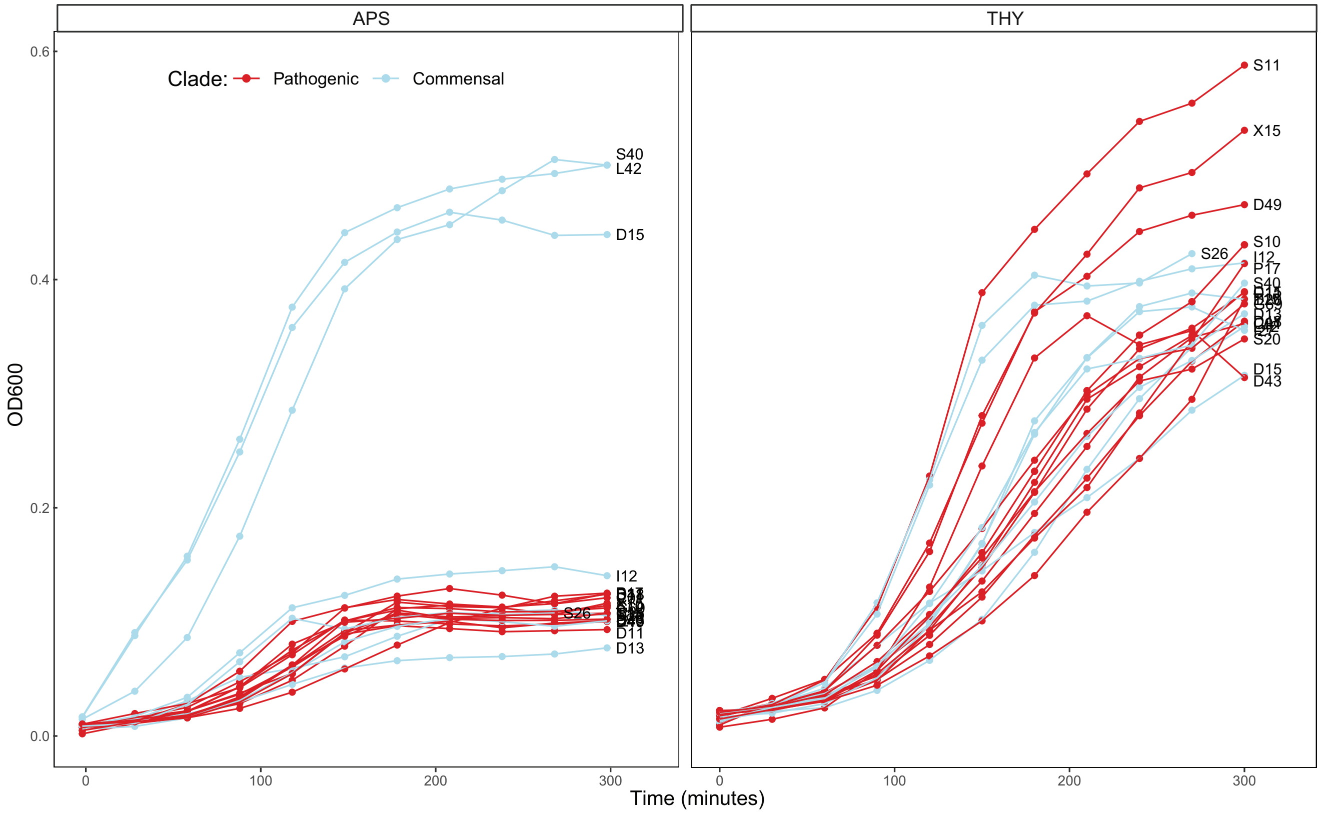

Figure S1: Growth curves. The strains were grown in 96-well plates with 200 µL total volume per well and incubated at 37 °C with 5% CO_2. Each point represents the mean value of the strain sampled in minimum 3 separate plates with 2 technical replicates per plate.

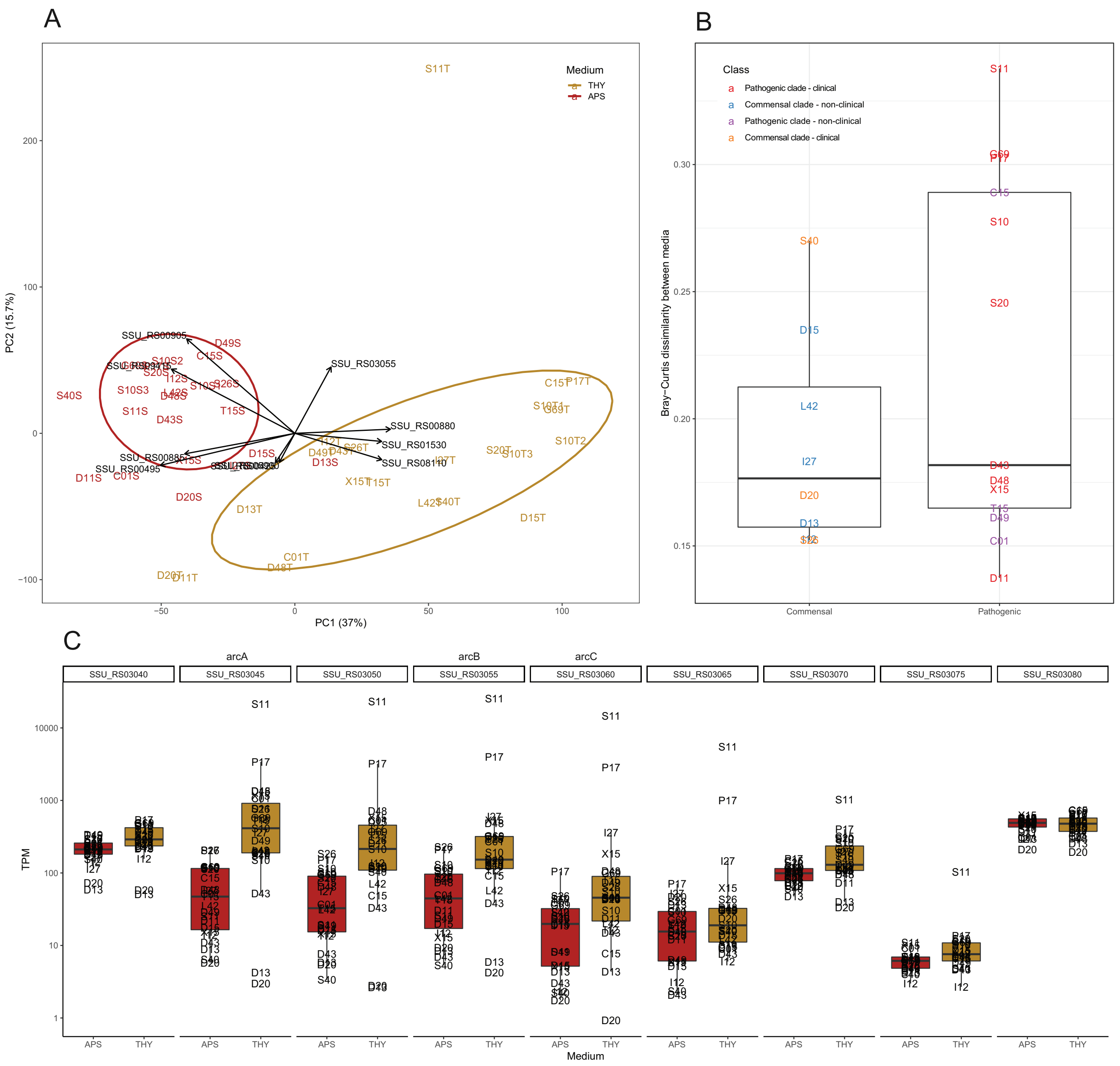

Figure S2: High ADS expression in S11 grown in THY. **A)** PCA on the core genome transcriptome of all strains shows sample S11T (strain S11 grown in THY) as an outlier. Each label represents a single sample, eclipses represent 75% confidence level, arrows show genes driving the separation of samples; samples in the direction the arrow is pointing have higher expression of the gene. **B)** Strain S11 also had the largest transcriptome difference between THY and APS cultures of the same strain (core genome transcriptome Bray-Curtis dissimilarity). **C)** Boxplot of expression (shown as log10(TPM) of *arcABC* and adjacent genes.

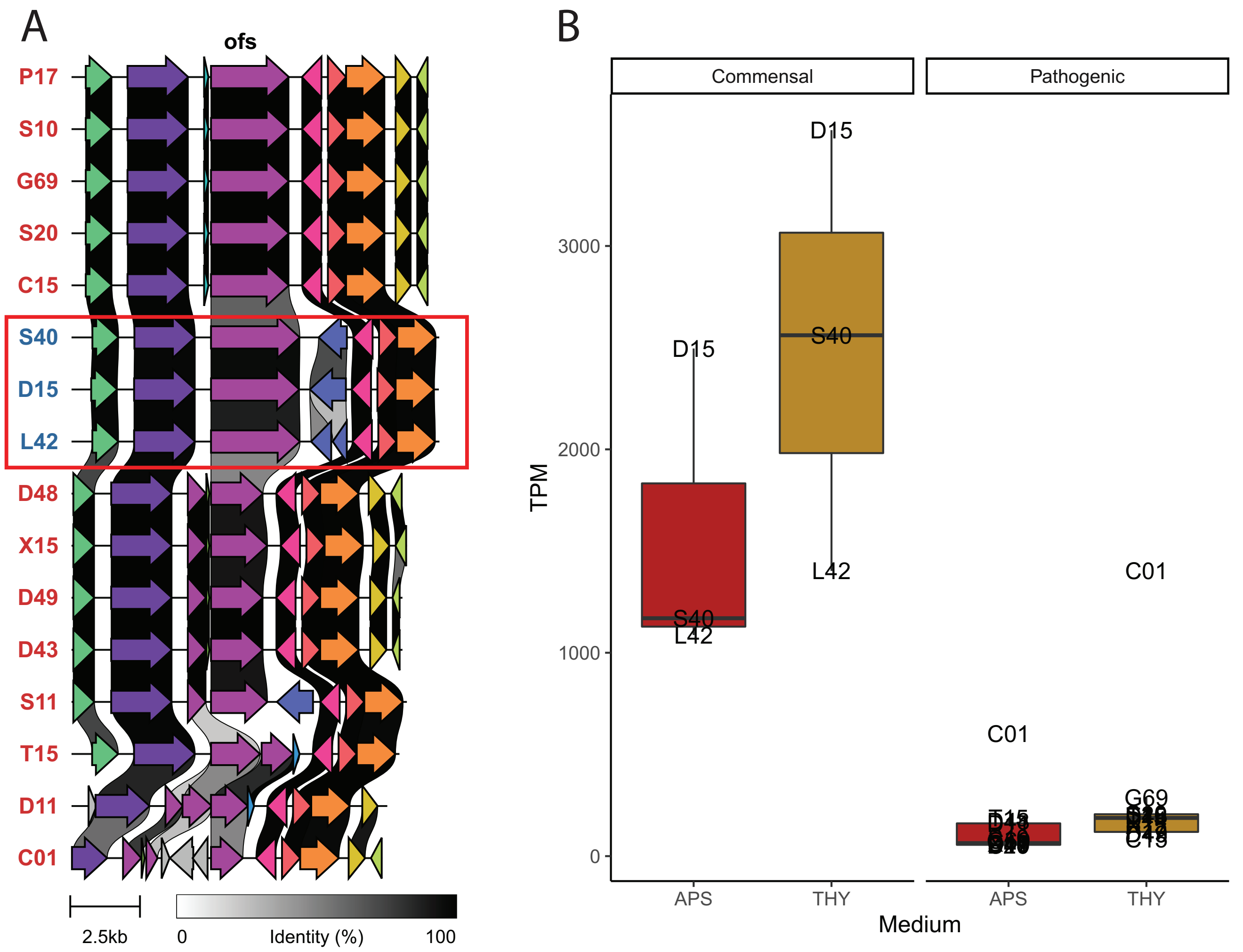

Figure S3: Opacification factor of serum (*ofs*). **A)** *ofs* occurs in different (suggested pseudogene) variants, but in similar genomic context. The 3 strains with high opaficification activity in serum (red box) share a similar variant (although there is substantial nucleotide level variation between these 3 strains). **B)** The 3 commensals with *ofs* had high expression of the gene. The other commensal strains did not have any detected *ofs* homologue.

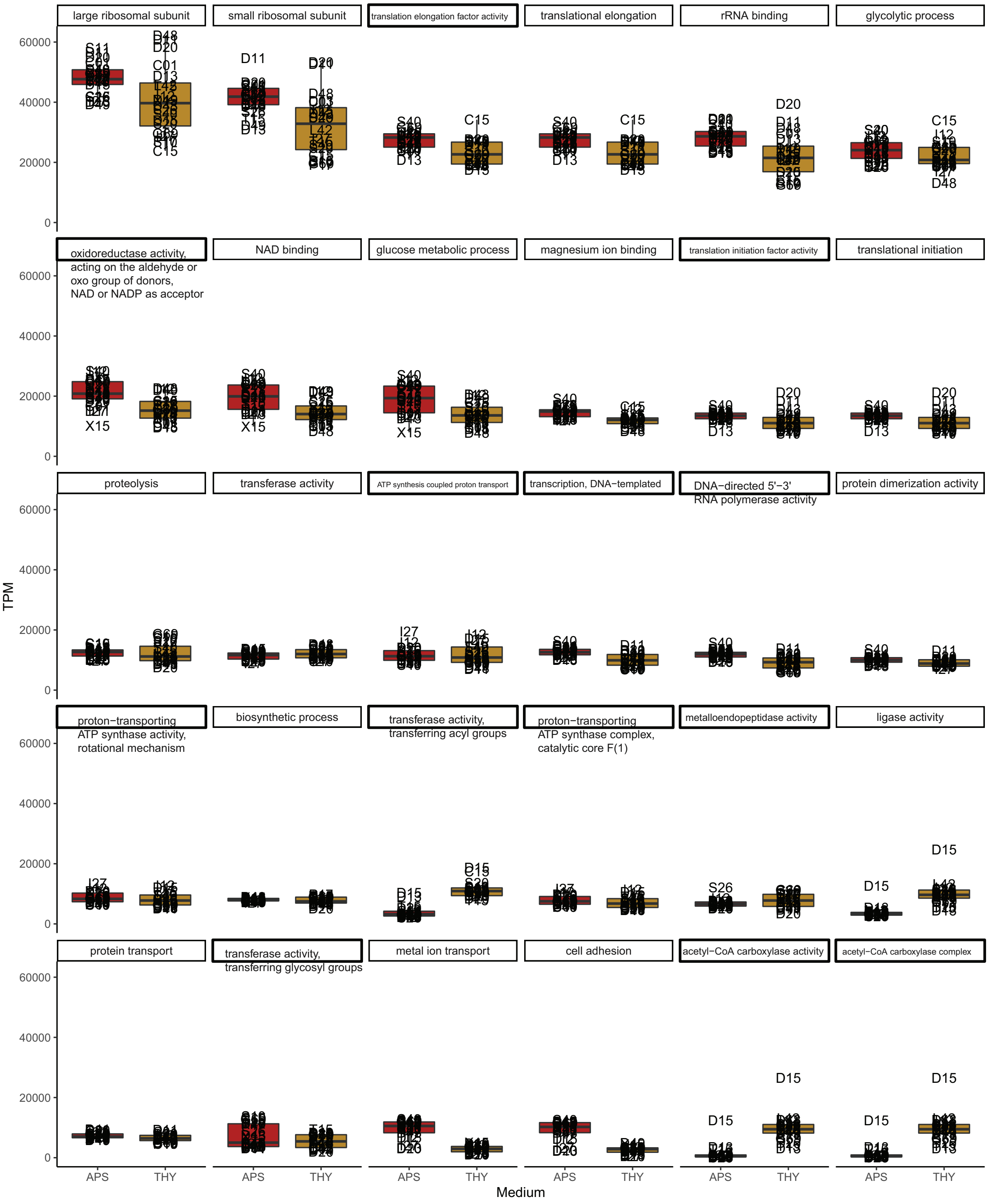

Figure S4: Boxplots showing expression of the overall most expressed GO terms in APS vs THY.

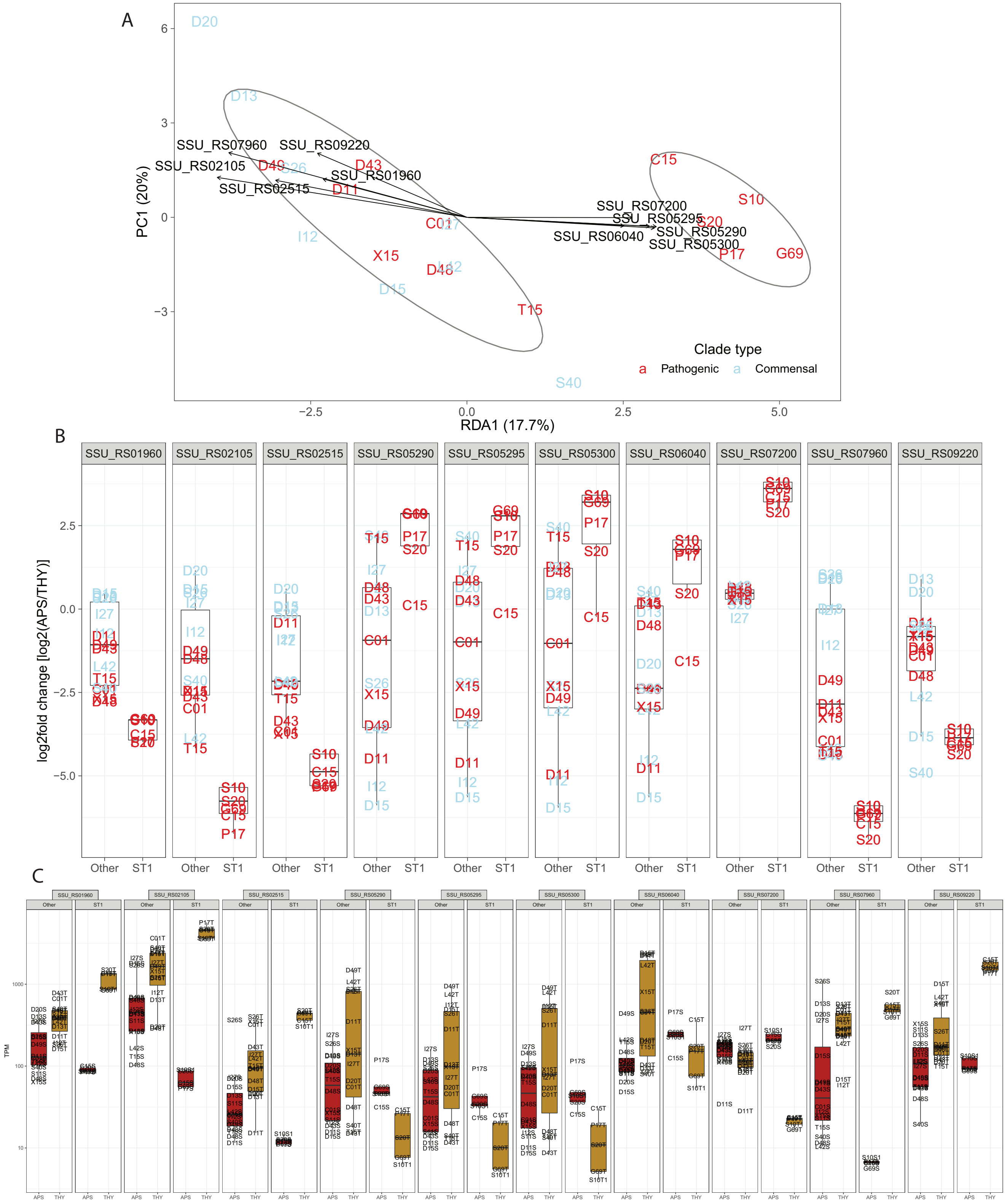

Figure S5: Genes strongly regulated between media in sequence type 1 strains. **A)** RDA on log2fold gene expression change constrained by ST1 clade strains vs all other strains. Each label represents a single sample, eclipses represent 75% confidence level, arrows show genes driving the separation of samples; samples in the direction the arrow is pointing have higher expression of the gene. **B)** Boxplot of the log2fold change of the 10 strongest drivers (genes) of the RDA1 axis. **C)** Boxplot of the TPM expression of the same 10 genes in APS and THY.

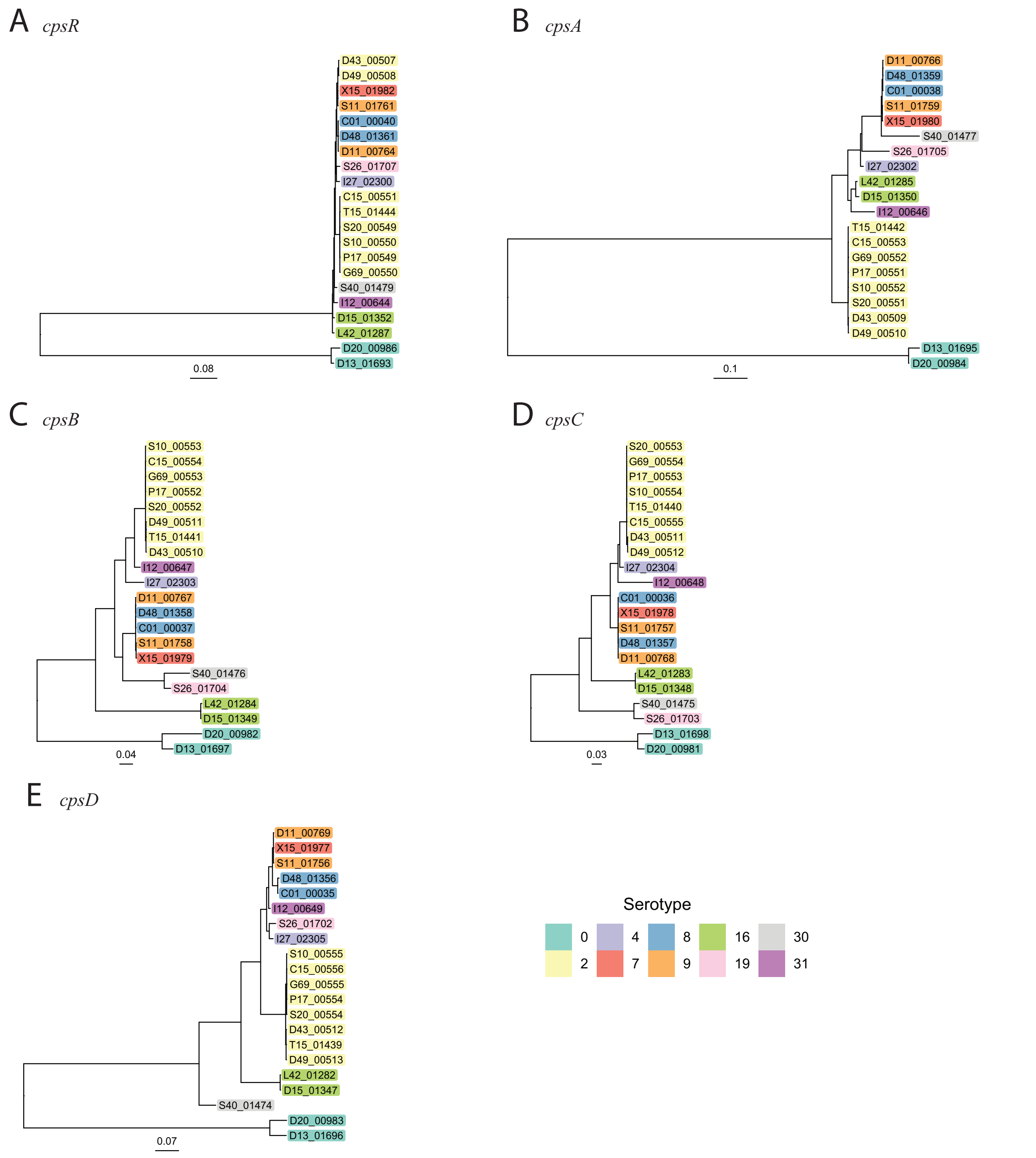

Figure S6: Maximum likelihood trees of *cps* genes (DNA). **A)** SSU_RS02755, *cpsR/gntR/orf2Y* **B)** SSU_RS02765, *cpsA* **C)** SSU_RS02770, *cpsB* **D)** SSU_RS02775, cpsC **E)** SSU_RS02780, *cpsD*. Tip labels: [Strain]_[gene number]. Except from outliers D13 and D20, *cpsR* is more conserved than *cpsABCD*. Diverged *cpsR* copies in strains D43 and D49 may underlie different *cps* regulation compared to other serotype 2 strains.

```
C15        ..MKKYVDTGHRKRGKSRRYHCSKDTVKAMKYNKYAVKSGYYDRDDHTVNTDYDRCKSGRNYNYYHGASSVSMDYHVYTKKDVTAGSAYT  88
G69        ..MKKYVDTGHRKRGKSRRYHCSKDTVKAMKYNKYAVKSGYYDRDDHTVNTDYDRCKSGRNYNYYHGASSVSMDYHVYTKKDVTAGSAYT  88
P17        ..MKKYVDTGHRKRGKSRRYHCSKDTVKAMKYNKYAVKSGYYDRDDHTVNTDYDRCKSGRNYNYYHGASSVSMDYHVYTKKDVTAGSAYT  88
S10        ..MKKYVDTGHRKRGKSRRYHCSKDTVKAMKYNKYAVKSGYYDRDDHTVNTDYDRCKSGRNYNYYHGASSVSMDYHVYTKKDVTAGSAYT  88
S20        ..MKKYVDTGHRKRGKSRRYHCSKDTVKAMKYNKYAVKSGYYDRDDHTVNTDYDRCKSGRNYNYYHGASSVSMDYHVYTKKDVTAGSAYT  88
T15        ..MKKYVDTGHRKRGKSRRYHCSKDTVKAMKYNKYAVKSGYYDRDDHTVNTDYDRCKSGRNYNYYHGASSVSMDYHVYTKKDVTAGSAYT  88
S40        ..MKKYVDTGHRKRGKSRSYHCSKDTVKAMKYNKYAVKSGYYDRDDHTVNADYDRCNSGRNYNYYHGASSVSMDYHVYTKKDVTAGSAYT  88
I12        ..MKKYVDTGHRKRGKSRSYHCSKDTVKAMKYNKYAVKSGYYDRDD.TVNADYDRCNSGRNYNYYHGASSVSMDYHVYTKKDVTAGSAYT  87
D15        ..MKKYVDTGHRKRGKSRSYHCSKDTVKAMKYNKYAVKSGYYDRDDHTVNADYDRCNSGRNYNYYHGASSVSMDYHVYTKKDVTAGSAYT  88
L42        ..MKKYVDTGHRKRGKSRSYHCSKDTVKAMKYNKYAVKSGYYDRDDHTVNADYDRCNSGRNYNYYHGASSVSMDYHVYTKKDVTAGSAYT  88
S26        ..MKKYVDTGHRKRGKSRSYHCSKDTVKAMKYNKYAVKSGYYDRDDHTVNADYDRCNSGRNYNYYHGASSVSMDYHVYTKKDVTAGSAYT  88
D43        ..MKKYVDTGHRKRGKSRRYHCSKDTVKAMKYNKYAVKSGYYDRDDHTVNADYDRCNSGRNYNYYHGASSVSMDYHVYTKKDVTAGSAYT  88
D49        ..MKKYVDTGHRKRGKSRRYHCSKDTVKAMKYNKYAVKSGYYDRDDHTVNADYDRCNSGRNYNYYHGASSVSMDYHVYTKKDVTAGSAYT  88
X15        ..MKKYVDTGHRKRGKSRSYHCSKDTVKAMKYNKYAVKSGYYDRDDHTVNADYDRCNSGRNYNYYHGASSVSMDYHVYTKKDVTAGSAYT  88
I27        ..MKKYVDTGHRKRGKSRSYHCSKDTVKAMKYNKYAVKSGYYDRDDHTVNADYDRCNSGRNYNYYHGASSVSMDYHVYTKKDVTAGSAYT  88
C01        ..MKKYVDTGHRKRGKSRSYHCSKDTVKAMKYNKYAVKSGYYDRDDHTVNADYDRCNSGRNYNYYHGASSVSMDYHVYTKKDVTAGSAYT  88
D11        ..MKKYVDTGHRKRGKSRSYHCSKDTVKAMKYNKYAVKSGYYDRDDHTVNADYDRCNSGRNYNYYHGASSVSMDYHVYTKKDVTAGSAYT  88
D48        ..MKKYVDTGHRKRGKSRSYHCSKDTVKAMKYNKYAVKSGYYDRDDHTVNADYDRCNSGRNYNYYHGASSVSMDYHVYTKKDVTAGSAYT  88
S11        ..MKKYVDTGHRKRGKSRSYHCSKDTVKAMKYNKYAVKSGYYDRDDHTVNADYDRCNSGRNYNYYHGASSVSMDYHVYTKKDVTAGSAYT  88
D13        ...MKYKDDGRDYKRGKSRSYCSKDTVKAMKYNKYAVKSGYYVDDR.DDDTDYDRCHSGRNYNYYHAGTTSVMDYHVYSRKDVTAGSAYS  86
D20        MSKYRDVDDGRDYKRGKSRSYCSKDTVKAMKYNKYAVKSGYYVDDH.DDDTDYDRCHSGRNYNYYHAGTTSVMDYHVYSRKDVTAGSAYS  89
consensus    *****!*!***********!!!!!!!!!!!!!!!!!!!!!**!******!!!!!*!!!!!!!!!******!!!!!!**!!!!!!!!!*

C15        MT.AGKTNTYSRMRHGYTRNDG...DSTGKKYTRHNGSTYDATKTAVKAKYDVYDDYADDSSHSHYDTDNRVYKSTTARGA.SNRDKHKS 173
G69        MT.AGKTNTYSRMRHGYTRNDG...DSTGKKYTRHNGSTYDATKTAVKAKYDVYDDYADDSSHSHYDTDNRVYKSTTARGA.SNRDKHKS 173
P17        MT.AGKTNTYSRMRHGYTRNDG...DSTGKKYTRHNGSTYDATKTAVKAKYDVYDDYADDSSHSHYDTDNRVYKSTTARGA.SNRDKHKS 173
S10        MT.AGKTNTYSRMRHGYTRNDG...DSTGKKYTRHNGSTYDATKTAVKAKYDVYDDYADDSSHSHYDTDNRVYKSTTARGA.SNRDKHKS 173
S20        MT.AGKTNTYSRMRHGYTRNDG...DSTGKKYTRHNGSTYDATKTAVKAKYDVYDDYADDSSHSHYDTDNRVYKSTTARGA.SNRDKHKS 173
T15        MT.AGKTNTYSRMRHGYTRNDG...DSTGKKYTRHNGSTYDATKTAVKAKYDVYDDYADDSSHSHYDTDNRVYKSTTARGA.SNRDKHKS 173
S40        MT.AGKTNT.YSRMHGYTRDDG...DSTGKKYTRHNGSTYDATKTAVKAKYNVYDDYADDSSHSHYDTDNRVYKSTTARGASNRYTKHKS 173
I12        MT.AGKTNTYSRMRHGYTRDDG...NSTGKKYTRHNGSTYDATKTAVKAKYDVYDDYADDSSHSHYDTDNHVYKSTTARGASNRDTKHKS 173
D15        MT.AGKTNTYSRMRHGYTRDDG...DSTGKKYTRHNGSTYDATKTAVKAKYDVYDDYADDSSHSHYDTDNRVYKSTTARGASNRDTKHKS 174
L42        MT.AGKTNTYSRMRHGYTRDDG...DSTGKKYTRHNGSTYDATKTAVKAKYDVYDDYADDSSHSHYDTDNRVYKSTTARGASNRDTKHKS 174
S26        MTWAGKTNTYSRMRHGYTRDDG...DSTGKKYTRHNGSTYDATKTAVKAKYDVYDDYADDSSHSHYDTDNRVYKSTTARGASNHDTKHKS 175
D43        MT.AGKTNTYSRMRHGYTRDDG...DSTGKKYTRHNGSTYDATKTAVKAKYDVYDDYADDSSHSHYDTDNRVYKSTTARGASNRDTKHKS 174
D49        MT.AGKTNTYSRMRHGYTRDDG...DSTGKKYTRHNGSTYDATKTAVKAKYDVYDDYADDSSHSHYDTDNRVYKSTTARGASNRDTKHKS 174
X15        MT.AGKTNTYSRMRYGYTRDDG...DSTGKKYTRHNGSTYDATKTAVKAKYDVYDDYADDSSHSHYDTDNRVYKSTTARGASNRDTKHKS 174
I27        MT.AGKTNTYSRMRHGYTRDDG...DSTGKKYTRHNGSTYDATKTAVKAKYDVYDDYADDSSHSHYDTDNRVYKSTTARGASNRDTKHKS 174
C01        MT.AGKTNTYSRMRHGYTRDDG...DSTGKKYTRHNGSTYDATKTAVKAKYDVYDDYADDSSHSHYDTDNRVYKSTTARGASNRDTKHKS 174
D11        MT.AGKTNTYSRMRHGYTRDDG...DSTGKKYTRHNGSTYDATKTAVKAKYDVYDDYADDSSHSHYDTDNRVYKSTTARGASNRDTKHKS 174
D48        MT.AGKTNTYSRMRHGYTRDDG...DSTGKKYTRHNGSTYDATKTAVKAKYDVYDDYADDSSHSHYDTDNRVYKSTTARGASNRDTKHKS 174
S11        MT.AGKTNTYSRMRHGYTRDDG...DSTGKKYTRHNGSTYDATKTAVKAKYDVYDDYADDSSHSHYDTDNRVYKSTTARGASNRDTKHKS 174
D13        MD.GDRTHSTYTRMRGYTRSMGDDRDKTGTKYTRHNG.TSYDRKTMRVAKRYVYDDYA.DDGNRHYDKNDVVYKSSTARGAVSDAT.YKG 172
D20        D..GDRTHSTYTRMRGYTRSMG.DDRGTGTKYTRHNG.TSYDRKTMRVAKRYVYDDYA.DDGNRHYDKNDVVYKSSTARGA.VSNATYKG 173
consensus  ** ***!********!!!!**!    **!!*!!!!!!!*!****!!***!!**!!!!!!*!****!!!****!!!!*!!!!!*******!*

C15        DYDTNMKASYDNGMARNTHHHYHAWNKKDCKYANYRSKGSVTSKGSSHMGKCYYSGKADDADKVRYN...  240
G69        DYDTNMKASYDNGMARNTHHHYHAWNKKDCKYANYRSKGSVTSKGSSHMGKCYYSGKADDADKVRYN...  240
P17        DYDTNMKASYDNGMARNTHHHYHAWNKKDCKYANYRSKGSVTSKGSSHMGKCYYSGKADDADKVRYN...  240
S10        DYDTNMKASYDNGMARNTHHHYHAWNKKDCKYANYRSKGSVTSKGSSHMGKCYYSGKADDADKVRYN...  240
S20        DYDTNMKASYDNGMARNTHHHYHAWNKKDCKYANYRSKGSVTSKGSSHMGKCYYSGKADDADKVRYN...  240
T15        DYDTNMKASYDNGMARNTHHHYHAWNKKDCKYANYRSKGSVTSKGSSHMGKCYYSGKADDADKVRYN...  240
S40        DYDTNMKASYDNGMARNTHHHYHAWNKKDCKYANYRSKGSVTSKGSSHMGKCYYSGKADDADKVRYN...  240
I12        DYDTNMKASYDNGMARNTHHHYHA.WNKKDCKYANYRSKGSVTSKGSSHMGKCYYSGKADDADKVRYN...  239
D15        DYDTNMKASYDNGMARNTHHHYHAWNKKDCKYANYRSKGSVTSKGSNHMGKCYYSGKADDADKTRYN...  241
L42        DYDTNMKASYDNGMARNTHHHYHAWNKKDCKYANYRSKGSVTSKGSNHMGKCYYSGKADDADKVRYN...  241
S26        DYDTNMKASYDNGMARNTHHHYHAWNKKDCKYANYRSKGSVTSKG.SSHMKCYYSGKADDADKVRYSRMN  244
D43        DYDTNMKASYDNGMARNTHHHYHAWNKKDCKYANYRSKGSVTSKG.SSHMKCYYSGKADDADKVRYN...  240
D49        DYDTNMKASYDNGMARNTHHHYHAWNKKDCKYANYRSKGSVTSKG.SSHMKCYYSGKADDADKVRYN...  240
X15        DYDTNMKASYDNGMARNTHHHYHAWNKKDCKYANYRSKGSVTSKG.SSHMKCYYSGKADDADKVRYN...  240
I27        DYDTNMKASYDNGMARNTHHHYHAWNKKDCKYTNYRSKGSVTSKG.SSHMKCYYSGKADDADKVRYN...  240
C01        DYDTNMKASYDNGMARNT.HHYHAWNKKDCKYANYRSKGSVTSKG.SSHMKCYYSGKADDADKVRYN...  239
D11        DYDTNMKASYDNGMARNT.HHYHAWNKKDCKYANYRSKGSVTSKG.SSHMKCYYSGKADDADKVRYN...  239
D48        DYDTNMKASYDNGMARNT.HHYHAWNKKDCKYANYRSKGSVTSKG.SSHMKCYYSGKADDADKVRYN...  239
S11        DYDTNMKASYDNGMARNTHHHYHAWNKKDCKYANYRSKGSVTSKG.SSHMKCYYSGKADDADKVRYN...  240
D13        DYDTNMKASYDNGMARNTRSNVDKKYAYYVVRDDNRTARGRHRNG...........TAYRHHDTKSTYKD  231
D20        DYDTNMKASYDNGMARNTRSNVNKKYAYYVVRDDNRTARGRYRNG...........TAYRHHDTKSTYKD  232
consensus  !!!!!!!!!!!!!!!!!!*****************!********!************!*********
```

X| non-conserved
X ≥50% conserved

Figure S7: Multiple sequence alignment of *cpsR* (SSU_RS02755) protein sequences. Strains D43 and D49 do not share sequence similarity with other serotype 2 strain, except from a R/S SNP in position 17.

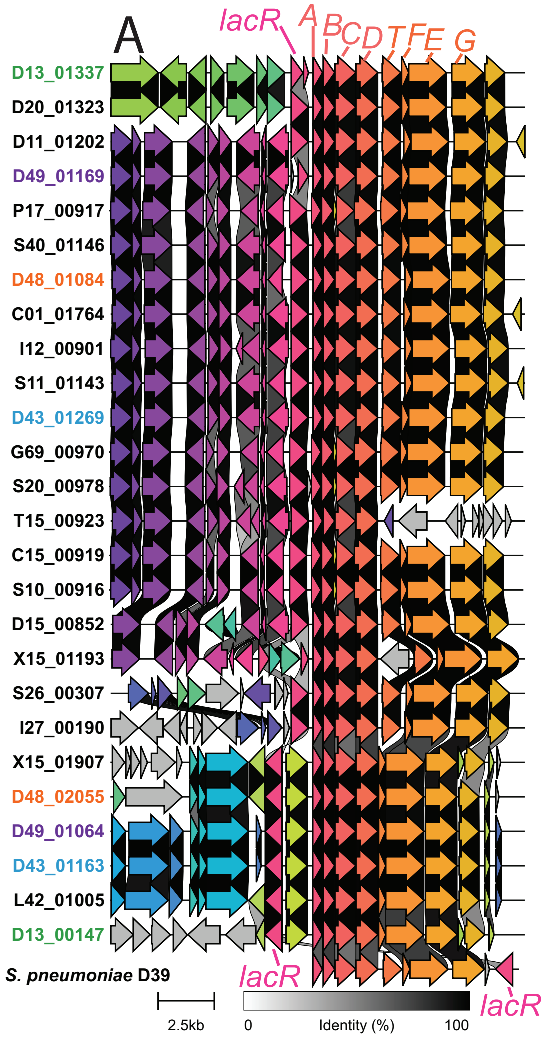

B

	THY				APS			
	lacR	*lacA*	*lacB*	*lacT*	*lacR*	*lacA*	*lacB*	*lacT*
C01	29.9	550.2	574.4	835.4	6.8	13.3	18.8	10.3
C15	19.6	471.6	258.1	111.4	13.3	13.3	6.4	6.7
D11	118.5	69.2	126.9	122.9	7.5	0.8	0.2	5.1
D13	418.8	265.0	321.3	51.7	508.4	208.1	280.2	95.3
D15	13.8	226.9	571.0	184.6	7.5	3.3	3.3	6.6
D20	137.1	1264.1	1352.5	346.5	59.1	2.0	2.2	5.5
D43	122.3	218.9	89.4	60.5	25.8	23.6	17.3	4.0
D48	35.7	21.1	20.5	11.3	46.5	25.9	16.3	5.3
D49	74.4	34.3	32.8	3.1	107.5	11.3	9.1	3.8
G69	43.3	502.3	319.7	230.1	10.9	19.6	12.7	8.1
I12	13.6	9.8	19.2	9.3	16.5	9.5	27.2	11.0
I27	195.8	1021.7	2307.4	1555.6	208.0	6.4	7.4	6.8
L42	98.4	273.1	196.6	NA	14.3	7.4	6.1	NA
P17	9.0	19.1	17.3	49.4	10.8	13.6	10.5	4.2
S10	38.8	452.8	314.8	191.9	8.5	13.1	11.7	5.2
S11					6.8	28.6	56.5	16.8
S20	60.9	738.0	562.5	247.7	7.0	28.0	16.4	5.2
S26	195.2	201.9	539.0	300.7	83.2	6.0	9.9	17.8
S40	9.3	3.7	8.4	10.2	11.6	9.0	9.1	6.1
T15	21.5	107.6	105.6	31.2	8.1	6.4	6.6	5.7
X15	74.2	140.9	134.1	3.4	44.3	19.1	19.2	1.8

Figure S8: Divergent *lac* gene cluster expression in strain D13. **A)** Several variants of the *lac* gene cluster are carried by the strains, and these may occur alone or two (at different locations) in the same genome. D13 has 2 *lac* gene clusters, which both feature inverted or pseudogene *lacR* copies (although other strains sharing the inverted variant had exopressino as expected). Genomes with several copies highlighted in color. Annotation based on Afzal et. al. 2014 (https://doi.org/10.1128/AEM.01370-14) and *S. pneumoniae* D39 assembly CP000410.2. **B)** *lacR* is highly expressed in D13, but *lacA* and *lacB* (and *lacT)* are nonetheless highly expressed in both media. P1/7 has unexpectly low *lac* expression compared to other ST1 strains.

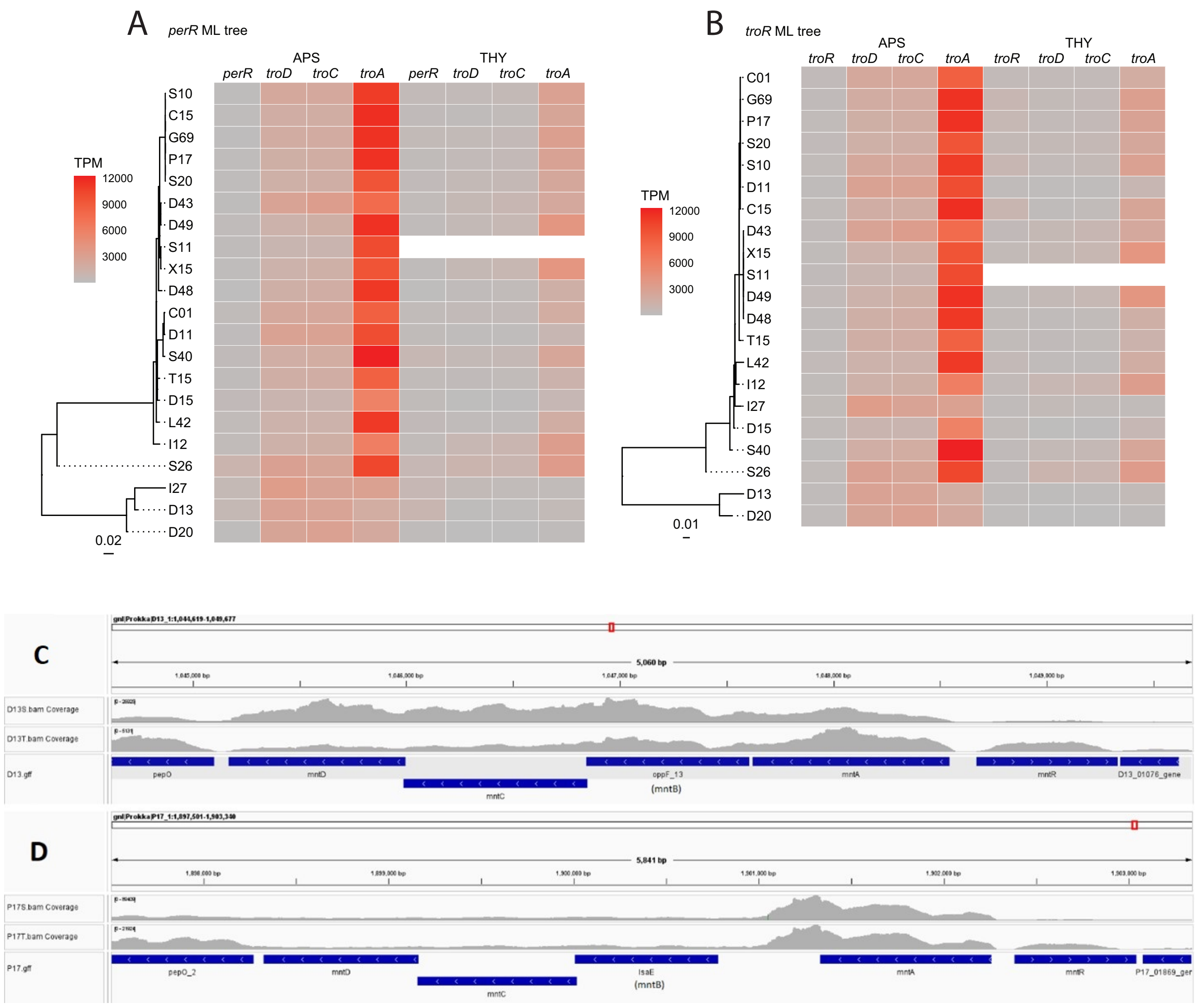

Figure S9: Gene expression of the *tro*/*mnt* gene cluster. A) ML tree of *perR* with *tro* expression heatmap. B) ML tree of *troR* with *tro* expression heatmap. C) Raw RNA-seq coverage of the *tro* gene cluster in strain D13 (visualized in IGV, https://igv.org). D) Raw RNA-seq coverage of the *tro* gene cluster in strain P1/7.
